# Mucopolysaccharidosis IIIB and mild skeletal anomalies: coexistence of *NAGLU* and *CYP26B1* missense variations in the same patient in a Chinese family

**DOI:** 10.1186/s12881-018-0562-4

**Published:** 2018-04-02

**Authors:** Jinliang Li, Han Xie, Yuwu Jiang

**Affiliations:** Department of Pediatrics, Peking University First Hospital, Beijing, 100034 China

**Keywords:** Mucopolysaccharidosis IIIB, *NAGLU*, *CYP26B1*, Novel pathogenic variation, Lysosomal storage diseases

## Abstract

**Background:**

Sanfilippo type B syndrome (mucopolysac-charidosis type IIIB; MPS IIIB) is an autosomal recessive lysosomal storage disorder. It is caused by a critically reduced α-2-acetamido-2-deoxy-D-glucoside acetamidodeoxy glucohydrolase (α-*N*-acetylglucosaminidase or NAGLU) activity. Recently, an autosomal recessive disorder of skeletal dysplasia associated with *CYP26B1* was reported in three families, in which the patients were all homozygous variations. However, the co-occurrence of two rare diseases in a person is very rare. Here, we reported one patient with two novel pathogenic missense variations in *NAGLU* and *CYP26B1*.

**Case presentation:**

We found an infant with biallelic variation both in *NAGLU*-compound heterozygous c.1843C > T (p. R615C) and c.1224C > A (p. H408Q) as well as in *CYP26B1*-compound heterozygous c.529G > A (p. E177K) and c.525C > A (p. H175Q). All variations were novel but predicted pathogenicity according to American College of Medical Genetics and Genomics (ACMG) guidelines. The main phenotypes of the infant were quite different from those previously reported, and some were combinations of the two rare diseases, including epilepsy, early onset epileptic encephalopathy, hypermyotonia, skull deformity, dilatation of the lateral ventricles and premature closure of fontanel. His NAGLU enzyme activity was significantly decreased.

**Conclusions:**

*NAGLU* and *CYP26B1* mutations were related to MPS IIIB and skeletal dysplasia, respectively. Here, we first reported the pathogenic mutations of two genes concurrent in one patient, which not only expands the phenotype and genotype spectra of *NAGLU* and *CYP26B1*, but more importantly indicates the possibility of simultaneous occurrence of two rare diseases in one patient. This interesting finding should be attributed to the use of whole exome sequencing (WES), which indicates that we should be aware of the importance of WES in diagnosing rare diseases.

## Background

Mucopolysaccharidosis type III (MPS III) or Sanfilippo syndrome includes five subtypes, each of which results from a specific impaired lysosomal enzyme, including sulfamidase (MPS IIIA); α-*N*-acetylglucosaminidase (NAGLU, MPS IIIB); heparan acetyl CoA *α*-glucosaminide *N*-acetyltransferase (MPS IIIC); *N*-acetylglucosamine 6-sulfatase (MPS IIID) and *N*-glucosamine 3-*O*-sulfatase (MPS IIIE) [1]. The pre-natal and early post-natal development are usually normal. The clinical features are similar in all the subtypes, which are characterised by progressive mental deterioration and behavioural problems with more or less prominent dysmorphic facial features.

Thus far, only two studies reported a syndrome of craniosynostosis, cranium bifidum, multiple skeletal abnormalities, and dilatation of the lateral and third ventricles due to biallelic variations in *CYP26B1* [2, 3]. In the two families reported in the first article described, a more severe phenotype led to intrauterine death in the three siblings, while in a single patient in the second family, the phenotype was milder to allow survival until five months of age.

In the present study, we reported a case with biallelic variations both in *NAGLU* and *CYP26B1*, whose main phenotypes were quite different from those previously reported, and with some combination of the two rare diseases, including epilepsy, early onset epileptic encephalopathy, hypertonia, skull deformity, dilatation of the lateral ventricles and premature closure of fontanel. Furthermore, *NAGLU* enzyme activity was significantly decreased.

## Case presentation

A 10-day-old new born was admitted to our unit due to limb hypertonia, pulling up with head lag, and repeated neonatal convulsions. From the second day after birth, he was presented with hypertonia, and four days later, he developed repeated convulsions.

**Fig. 1:**
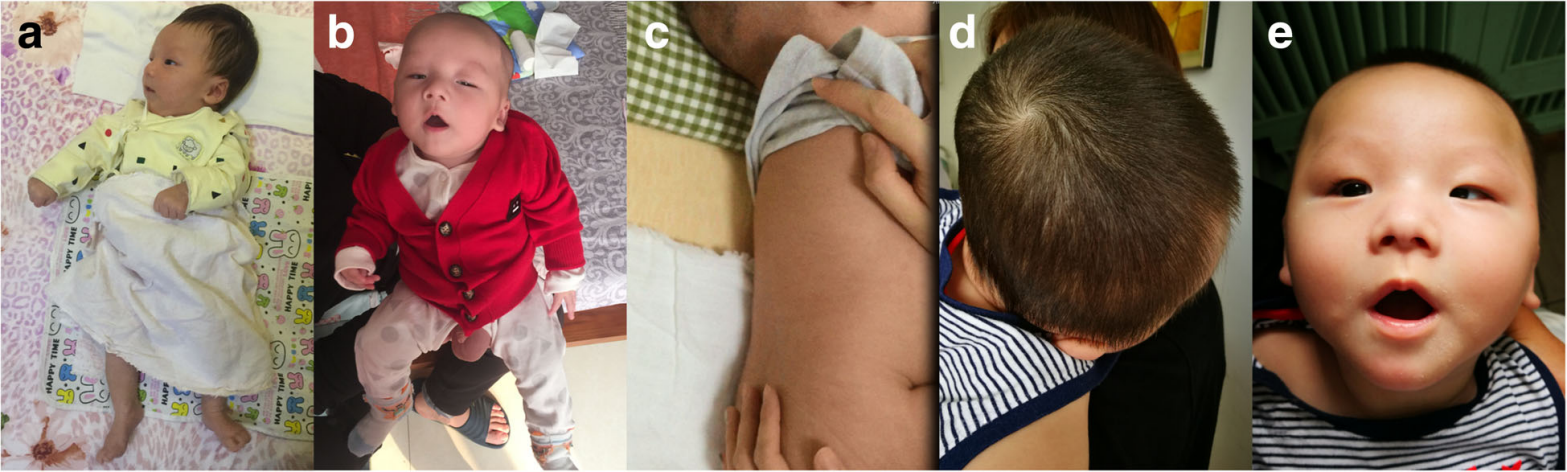
Serial photographs of the patient. **a** Hypertonia at one month old, **b** inguinal hernia and **c** kyphosis at 100 days old. **d** Skull deformity and **e** facial asymmetry at 8 months old

On physical examination, umbilical hernia, inguinal hernia, kyphosis, hypertonia and skull deformity were found (Figs. 1a, b, c). He did not show facial coarseness, hypertrichosis and other positive signs reported to be related to *NAGLU* and *CYP26B1* gene variation.

**Fig. 2:**
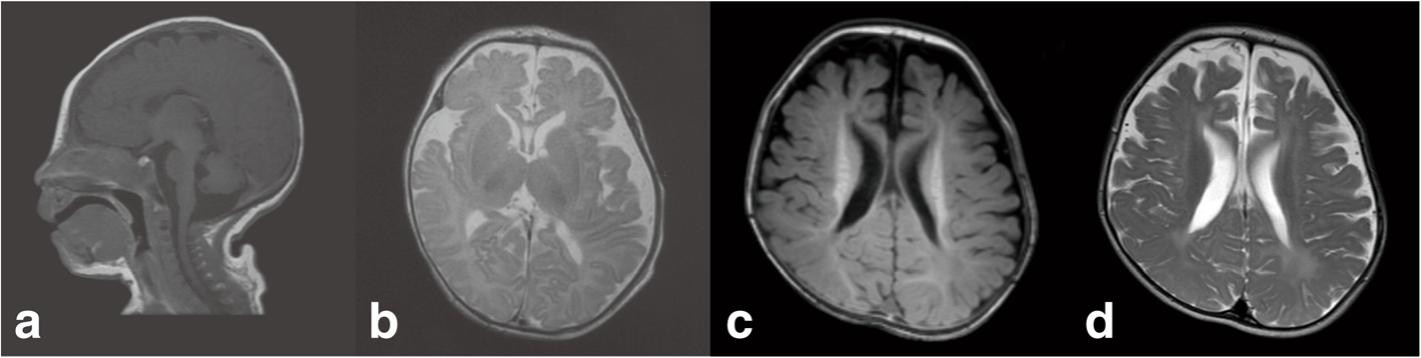
Head MRI of the siblings. **a**, **b** Head MRI of the sister at 25 days old showed skull deformity and asymmetrical bilateral structure. **c**, **d** Bilateral ventricle enlargement, asymmetry of brain structure, decreased white matter and thin corpus callosum of the proband at 8 months old

He was a full-term baby without any problem during his birth. He had an elder sister who died at the 30th day after birth. When the sister was still a foetus, her ultrasound examination indicated that her brain lateral ventricle was enlarged. She was naturally delivered and a full-term baby. From the 10th day after birth, she had lethargy and feeding difficulty but no seizure. Facial asymmetry was observed, and her brain magnetic resonance imaging (MRI) results indicated skull deformity, and the bilateral structure was asymmetrical (Figs. 2a, b).

After admission, the boy was treated with vitamin B6 and vitamin B12, but the treatment was ineffective. At the 16th day after birth, he was administered with Topamax. With the increase of the dosage, the convulsions decreased transiently. Unilateral facial muscles began to twitch at the 26th day after birth, which lasted several seconds every time, and the longest was 10 s. The convulsions and the facial twitches disappeared after the first month of life but spasms occurred. Levetiracetam and Vigabatrin were then gradually added.

Video-electroencephalograph showed gradual deterioration, followed by hypsarrhythmia and spasms (Table 1).

**Table 1:** Evolution of process of V-EEG

Age	V-EEG
11 days	Moderately abnormal neonatal EEG with moderately delayed maturity of electricity of the brain (alternate and discrete graphics during QS period). Multiple focal and interhemispheric sharp wave and slow wave activated asynchronously, which is primarily in the left temporal region. Unilateral or bilateral front head was distributed with intermittent theta rhythms.
39 days	Abnormal infant EEG with multiple focal spike waves, sharp waves, and irregular slow waves discharged non-synchronously, which mainly at the right central-parietal region. The lateral occipital areas showed intermittent low wave amplitude and fast activities. There were multiple focal episodes during waking and sleeping stages, and the suspected local myoclonus and spasms.
3 months and 3 days	Indicated multifocal spike waves, sharp waves, spike slow wave complex, sharp slow wave complex and slow waves which primarily distributed at the frontal and temporal regions and detected multiple spasms during waking and sleeping stages.
5 months and 18 days	Hypsarrhythmia which were primarily at occiput

The routine and biochemical laboratory test results of cerebrospinal fluid (CSF) were normal. Microbe examinations of the CSF, urine and blood were negative. The screening test results for metabolic diseases in blood and urine were normal. Mitochondrial genes were normal. The infant was diagnosed with epilepsy and early onset epileptic encephalopathy (EOEE).

**Fig. 3:**
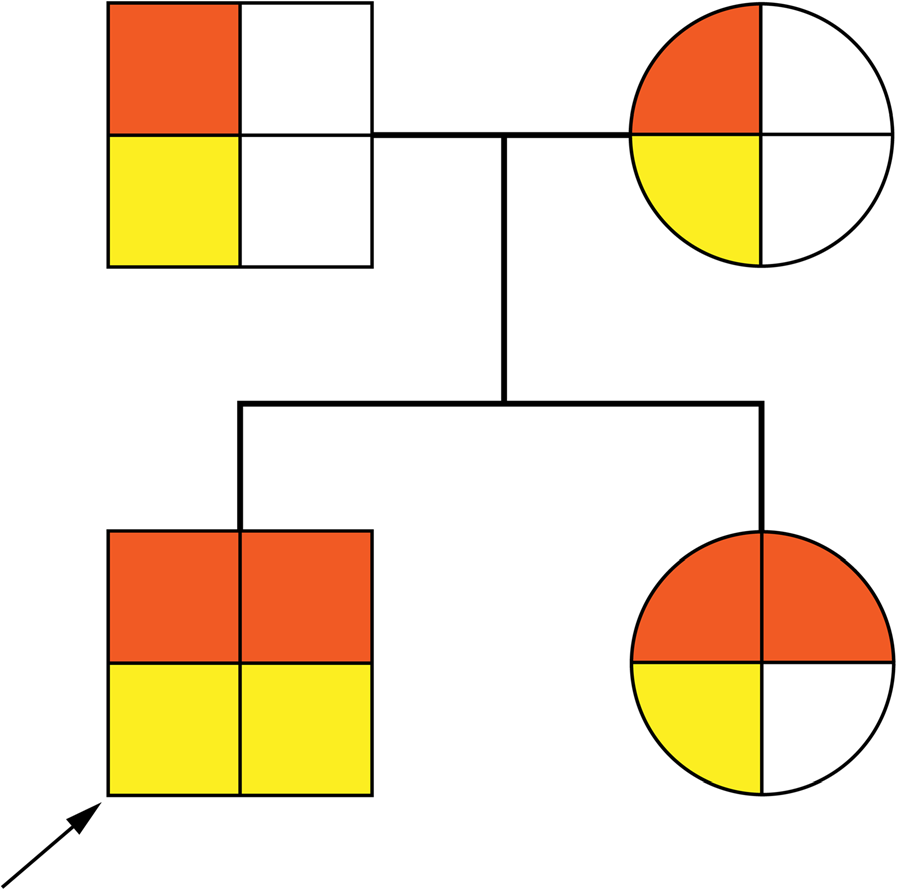
Pedigree of the family. Arrow indicates the proband. Brown represents variants of *CYP26B1*, and yellow represents variants of *NAGLU*. The variants c.1843C > T (p.R615C) in *NAGLU* and c.529G > A (p.E177K) in *CYP26B1* were paternal, while the variants c.1224C > A (p.H408Q) in *NAGLU* and c.525C > A (p.H175Q) in *CYP26B1* were maternal

The WES in family trios was conducted. The amplified DNA was captured using GenCap exome capture kit (MyGenostics). The enrichment libraries were sequenced on Illumina HiSeq X ten sequencer for paired read at 150 bp. Finally, we found the compound heterozygous c.1843C > T (p.R615C) and c.1224C > A (p.H408Q) variants in *NAGLU.* Through verification by Sanger sequencing, we confirmed that c.1843C > T was paternal and c.1224C > A was maternal (Fig. 3). The variations of the two sites were both novel. According to ACMG guidelines [4], both the variations were categorized to be “pathogenic”(c.1843C > T met PS3, PM1, PM2, PM3, PP3 and PP4; c.1224C > A met PS3, PM1, PM2, PM3, PP4).

At the same time, we found another compound heterozygous c.529G > A (p.E177K) and c.525C > A (p.H175Q) variants in *CYP26B1.* Through verification by Sanger sequencing, we confirmed that c.529G > A was paternal and c.525C > A was maternal (Fig. 3). Both the variations were categorized to be “likely pathogenic”(c.529G > A met PM2, PM3, PP1, PP2 and PP4; c.525C > A met PM2, PM3, PP1, PP2 and PP4) according to ACMG guidelines. Fortunately, the sister’s blood specimen is available, and her *CYP26B1* were compound heterozygous, which was the same as the younger brother. However, the sister had only one heterozygous mutation of *NAGLU*. No pathogenic mutation of known EOEE-related genes had been found in this trios WES.

Then we measured the lysosomal enzyme activity in this boy and found that *NAGLU* enzyme activity was significantly decreased (0.3 nmol/g/min, reference range, 7.3–32.4 nmol/g/min).

Now, the boy is 8 months old, and his weight is 9.25 kg. The height and head circumference seem normal. However, he experienced frequent epileptic spasms and repeated respiratory infection are happening to him. Anterior fontanelle has been closed. Facial asymmetry and skull deformity were apparent (Figs. 1d, e). Cry is rare and weak, and sleep disturbance is obvious. The re-examined brain MRI (Figs. 2c, d) showed bilateral ventricle enlargement, asymmetry of brain structure, decreased white matter and thin corpus callosum.

## Discussion and conclusions

Five subtypes of MPS III and their clinical features are similar among all the subtypes. Pre-natal and early stages of post-natal development are usually normal. The severe phenotype is usually more common, and it can be divided into three phases [5]. The first phase may begin between the ages of 1 and 3 years, and the patients mainly manifested as delayed cognitive development and/or aggressive behavioural problems, as well as hindered speech development [6]. The second phase starts at approximately 3–4 years of age and is followed by progressive cognitive deterioration and the emergence of behavioural difficulties and sleep disturbances [7]. The third phase begins, usually in the teenage years, with the onset of severe dementia and motor function decline. All motor functions start to decline, and the patient deteriorates into a vegetative state. Death usually occurs early in the third decade of life. However, patients with attenuated phenotypes surviving for 69 years were reported [8]. The patient in our study indicates that the age of onset of MPS IIIB may be early in the neonatal period, and early onset epileptic encephalopathy may have been related to *NAGLU* biallelic mutations because no other mutation was found in trio WES analysis, including the EOEE-related genes, and epilepsy did not occur in the older sister who had only one heterozygous mutation of *NAGLU*.

Diagnostic delay in the population of MPS III is very common, particularly in patients with a slowly progressing or mild phenotype. The boy in this study can obtain an earlier diagnosis because of the help of Next-generation sequencing (NGS), especially WES. This study found not only the variations of *NAGLU*, but also compound heterozygous variations of *CYP26B1*, which was associated with skeletogenesis. With the development of NGS technique, gene testing becomes more convenient. When encountering a rare disease of unknown origin, we should perform a comprehensive genetic test to confirm the diagnosis.

*NAGLU* contains six exons, and the cDNA coding for a 720-amino acid mature protein has six potential N-glycosylation sites [9]. No common pathogenic variations in MPS IIIB was observed, and most of the known pathogenic variations in MPS IIIB patients occur at low frequencies or not more than once [10]. Over 160 variations in *NAGLU* gene by far have been identified as disease-causing, and most of them were missense/nonsense type (HGMD Professional). The variations we found in *NAGLU* in this study were both novel. According to ACMG guidelines, both the variations were categorized to be “pathogenic”. Variations in *NAGLU* can cause MPS IIIB or Charcot–Marie–Tooth disease (CMT). However, the siblings in the present study had no any phenotypes of CMT disease. Moreover, NAGLU enzyme activity was significantly decreased. Although some unusual presentations were observed, such as neonatal epileptic seizure and very early developmental delay, according to the main phenotypes, enzyme activity assay and pathogenic *NAGLU* mutations, the diagnosis of MPS IIIB is definite.

*CYP26B1* encodes a cytochrome P450 enzyme responsible for the catabolism of retinoic acid (RA) in a very restricted fashion during embryonic development [3]. Both the deficiency and excess of RA have considerable impacts during vertebrate embryogenesis and organogenesis. Some studies showed that abnormalities of limb and craniofacial morphogenesis can result from exposure to exogenous RA at critical periods during foetal skeletal development [11]. In the three siblings and an unrelated Turkish patient with radiohumeral fusions and other skeletal and craniofacial anomalies, Laue et al. identified homozygous missense variations in the *CYP26B1* gene (R363L and S146P) [11]. The severe phenotypes of the three siblings led to intrauterine death, and the baby in the second family survived up to five months of age. However, the patient in another article survived into adulthood, and she had dilatation of brain ventricles [3]. In this study, compound heterozygous variations of *CYP26B1* were found in the two siblings. The phenotypes of the older sister exhibited facial asymmetry, encephalomalacia of the white matter, and asymmetrical bilateral structure. The proband of this study showed facial asymmetry, skull deformity, ventricle enlargement, asymmetry of brain structure and premature closure of fontanel. According to the malformations of the skull and the ACMG guidelines, those compound heterozygous variations of *CYP26B1* were probably pathogenic.

In summary, in this case, the phenotypes of MPS IIIB were developmental delay, sleep disturbance, epilepsy, repeated respiratory infections, navel hernia, inguinal hernia and decreased NAGLU enzyme activity; whereas, phenotypes related to variant *CYP26B1* included facial asymmetry, skull deformity, bilateral ventricle enlargement and asymmetry of brain structure, which were presented in both kids. Kyphosis, in this case, might be the common manifestation of both diseases.

The estimated incidence of MPS IIIB is very low and varied among the countries. Some studies found that MPS IIIA was the most frequent type in Northern Europe [10, 12, 13], whereas MPS IIIB is more frequent in Southern Europe [1, 14, 15]. The most frequent type in Chinese Taiwan was MPS IIIB, according to a study (0.28 per 100,000 live births) [16]. However, epidemiologic investigations about this disease in mainland China were not conducted. Skeletal dysplasia associated with the variations of *CYP26B1* might be rare, and only two related reports have been published.

Given the lack of a specific therapy, the present efforts are generally relieving strategies. However, promising therapies for MPS III are continuously being developed and evaluated, such as enzyme replacement therapy (ERT), gene therapy, genistein and substrate deprivation therapy. The ability for the enzyme NAGLU to be secreted from the lysosomes within cells has provided the basis for the foundation of therapies for MPS IIIB. However, the major obstacle of ERT is the inability of the enzyme to cross the blood brain barrier. The use of gene therapy is an extension of ERT as it attempts to introduce the coding sequence of the protein of interest into the cells of the patient using a viral vector, rather than synthesize it and purify it from another host [1].

The co-occurrence of two rare diseases in a patient is very rare. We diagnosed a patient with MPS IIIB due to compound heterozygous pathogenic variations of *NAGLU*, as well as skeletal dysplasia, associated with the compound heterozygous pathogenic variations of *CYP26B1.* We also found two novel missense variations in *NAGLU* and *CYP26B1* respectively. This report will provide a better understanding of MPS IIIB due to *NAGLU* variation and skeletal dysplasia associated with *CYP26B1*. The simultaneous discovery of two rare diseases in one patient reminded us to value the importance of WES in precisely diagnosing complex cases.

## Abbreviations

ACMG: American College of Medical Genetics and Genomics
CMT: Charcot–Marie–Tooth disease
CSF: Cerebrospinal fluid
ERT: Enzyme replacement therapy
MPS III: Mucopolysac–charidosis type III
MRI: Magnetic resonance imaging
NAGLU: α-N-acetylglucosaminidase
NGS: Next-generation sequencing
RA: Retinoic acid
WES: Whole exome sequencing
